# The effects of different dietary nutritional levels on meat quality, rumen microbiota, and muscle metabolomics in Tibetan Plateau yaks

**DOI:** 10.3389/frmbi.2025.1545689

**Published:** 2025-03-10

**Authors:** Shengchun Xu, Shuxiang Wang, Jiyuan Zhang, Xun Wang, Yingkui Yang, Shengsheng Li, Yang Xiang, Hongxin Nie, Yuming Li, Ziming Zeng, Shatuo Chai, Shujie Liu

**Affiliations:** ^1^ Qinghai Academy of Animal Husbandry and Veterinary Sciences in Qinghai University, Xining, Qinghai, China; ^2^ Key Laboratory of Plateau Grazing Animal Nutrition and Feed Science of Qinghai Province, Xining, Qinghai, China; ^3^ Yak Engineering Technology Research Centre of Qinghai Province, Xining, Qinghai, China; ^4^ New Hope Ecological Animal Husbandry Company Limited, Chengdu, Sichuan, China

**Keywords:** yak, dietary nutritional levels, meat quality, metabolomics, 16S rRNA

## Abstract

**Introduction:**

The nutritional level of the diet plays a crucial role in maintaining the balance of the yak rumen microbiota. To explore the relationship between dietary nutritional levels, the rumen microbiota, and muscle metabolites, we examined the characteristics of the yak rumen microbiota and muscle metabolome under different dietary nutritional levels.

**Methods:**

Randomly divide 24 yaks with similar body weights, [235.96 ± 12.46 kg], into three groups. These groups were subjected to three nutritional feeding levels: ad libitum feeding (AL), 70% of ad libitum intake (IR70), and 40% of ad libitum intake (IR40). When the yaks in the AL group gained 70 kg in body weight, they were slaughtered.

**Results:**

The results indicated that the ad libitum feeding group (AL) demonstrated superior edible meat quality in terms of Chroma L*, Chroma a*, and shear force, compared to the 70% intake group (IR70) and the 40% intake group (IR40). At the phylum level, the abundance of Patescibacteria was notably greater in the IR40 group compared to both the AL group and the IR70 group. At the genus level, the relative abundance of Succinimonas was higher in the AL group than in both the IR70 and IR40 groups. Untargeted metabolomics analysis revealed that the levels of metabolites such as 5-Methylcytosine, Cytosine, and Thymine were upregulated in the longissimus dorsi muscle of the AL group, which contributed to the enhancement of meat flavor. Furthermore, Spearman's correlation analysis revealed a notable relationship between the rumen microbiota and both meat quality and metabolite levels. pH45min is positively correlated with trans-Cinnamic acid. Methanobrevibacter exhibited a positive correlation with the concentration of 4-(Diethylamino)benzaldehyde, while Candidatus_Saccharimonas showed a positive correlation with the concentration of phenylacetylglycine.

**Discussion:**

This study provides scientific evidence for understanding the impact of different nutritional feeding conditions on yak meat quality, rumen microbiota, and related muscle metabolomic pathways. It also reveals the potential impact of these factors on meat flavor. These findings offer important reference information for optimizing yak husbandry management, improving the formation of beef flavor compounds, and understanding their regulatory mechanisms.

## 1 Introduction

Yaks have gained significant attention due to their adaptability to high altitudes and extreme environments (He et al., 2024). Their meat is known for its delicious flavor and firm texture, making it highly favored by consumers. However, due to the harsh climate in high-altitude pastoral areas, with a short grass-growing season and a long dry season, the forage available cannot meet the year-round feed needs of grazing yaks (West, 2015). This results in a growth pattern of yaks characterized by “fat in summer, strong in autumn, lean in winter, and dying in spring” (Shuli et al., 2024). These environmental factors lead to significant seasonal variations in both the yield and quality of yak meat (Guo et al., 2024). Nutrition, as the material basis for maintaining normal physiological functions and productivity in animals, directly influences the growth and development of yaks at different nutritional levels (Katongole and Yan, 2020), thereby affecting both the yield and quality of yak meat. Therefore, how to improve the nutritional supply for yaks to maintain stable growth throughout the year, while enhancing meat quality and yield, has become an issue that needs to be addressed.

The rumen, a vital part of the yak’s digestive system, serves as the main site for fibrous material digestion and plays an essential role in energy metabolism and microbial fermentation (Chen et al., 2025). The microbial community in the rumen, including bacteria, fungi, and archaea, plays a crucial role in the nutritional absorption and health of yaks. These microorganisms can convert forage into volatile fatty acids and microbial protein, thereby supplying the host with a significant portion of its energy and protein requirements (Omondi et al., 2024). Additionally, the fat content and fatty acid composition within yaks are significantly influenced by the nutritional level of the rumen and microbial metabolism (Pang et al., 2024), which directly affects the concentration and production of metabolites in the muscle.

The types and concentrations of metabolites in meat are important indicators for assessing meat flavor and palatability (Ramanathan et al., 2023). These metabolites determine the physiological characteristics of the muscle and also influence meat quality traits (Ueda et al., 2019). For example, the valine metabolite 3-hydroxyisobutyrate (3-HIB) promotes the uptake of fatty acids and lipid accumulation in skeletal muscle by activating endothelial fatty acid transporters (Zhang et al., 2021), thereby influencing the tenderness and juiciness of the meat (Alahakoon et al., 2016). Supplementation with high levels of isoleucine can increase the pH value of meat (pH at 24 hours post-mortem) and tends to reduce the drip loss from the longissimus dorsi muscle (Xu et al., 2020). Adding concentrate feed to the diets of grazing lambs helps lower the n-6/n-3 polyunsaturated fatty acid ratio in the muscle and improves carcass quality and fatty acid composition (Ramos et al., 2020). Furthermore, metabolomics studies can reveal how the nutritional components of feed affect the levels of amino acids and fatty acids in muscle (Guo et al., 2024), providing a basis for formulating scientific feeding strategies.

To date, there has been no unified standard regarding the nutritional requirements for yak husbandry, either domestically or internationally. In practice, the formulation of yak diets is often based on experience or by referring to the nutritional requirements for beef cattle (NRC, 2007). In-depth research to determine the appropriate dietary nutritional levels will not only provide a solid foundation for ruminant nutrition research but also hold significant theoretical and economic value for guiding ruminant production practices and establishing scientific and reasonable feeding standards for yaks. However, current research on how dietary nutritional levels influence the rumen microbiota and muscle metabolism in yaks, as well as the interactions between these factors, is still limited and requires further exploration and clarification. Therefore, this study hypothesizes that changes in dietary nutritional levels will affect the composition and function of the yak rumen microbiota, and that this impact may subsequently influence the metabolite levels in the muscles, ultimately leading to changes in meat quality characteristics. To verify this hypothesis, the study employed 16S rDNA sequencing and non-targeted metabolomics analysis methods to explore the specific effects of different nutritional levels on the yak rumen microbiota. At the same time, by systematically investigating the changes in the yak muscle metabolome, this study aims to uncover potential correlations between the rumen microbiota and muscle metabolites.

## 2 Materials and methods

This study has been approved by the Animal Protection and Utilization Committee of Qinghai University (Approval number: QHU20220915), and all handling of the yaks was conducted in accordance with ethical guidelines.

### 2.1 Experimental design

The experiment was conducted from September to December 2023 at the Plateau Modern Ecological Animal Husbandry Science and Technology Experimental Demonstration Park in Haiyan County, Haibei Prefecture, Qinghai Province. The experiment lasted for a total of 105 days, which included a 15-day pre-feeding phase and a 90-day main trial period.

A total of 24 healthy male yaks, each approximately four years old and weighing [(235.96 ± 12.46) kg], were selected and randomly assigned to three groups, with eight yaks per group, following a one-way variable design. The three groups were subjected to three nutritional feeding levels: ad libitum feeding (AL), 70% of ad libitum intake (IR70), and 40% of ad libitum intake (IR40). When the yaks in the AL group had gained 70 kg in body weight, they were slaughtered. The IR40 group served as the maintenance requirement group in the experimental design (target daily weight gain of 0 g/d), while the IR70 group was positioned between the ad libitum feeding group and the maintenance requirement group (target daily weight gain of 700 g/d). The feed intake of the two restricted feeding groups (IR70 and IR40) was adjusted based on the minimum feed intake of yaks in the ad libitum feeding group (AL group) from the previous day, ensuring that approximately 10% of the feed remained in the trough each day. None of the yaks underwent castration, and all received the necessary vaccinations before the start of the experiment. Each yak was assigned a unique identification number, and yaks in each group were housed in individual stalls and fed separately. They had free access to water and were fed twice daily, at 8:00 AM and 5:00 PM, with a total mixed ration provided. The diet was formulated according to the Chinese “Standards for Beef Cattle Feeding” (NY/T815-2004). Before and after the experiment, the yaks were weighed, and the dry matter intake was recorded. The nutritional composition and levels of the experimental diet are shown in **Table 1**.

**Table 1:** Basic diet composition and nutritional level (DM basis).

Feeds composition		Nutritional level	
Ingredients	Ratio (%)	Items	Content/(%)
Oats hay	50.00	Dry matter	89.47
Corn	22.68	Metabolic energy(M/kg)	10.98
Wheat	6.25	Crude protein(CP)	12.41
Wheat bran	6.43	Ether Extract(EE)	4.97
Rapeseed dregs	6.36	Neutral washing fiber(NDF)	37.67
Soybean meal	2.17	Acid detergent fiber(ADF)	23.32
Palm oil fat powder	2.11	Ca	0.45
CaHCO_3_	1.00	P	0.48
NaCl	1.00		
Premix^1^	2.00		
Total	100.00		

^1^Premixed ingredients provided VA 4 000 IU, VD 500 IU, VE 40 IU, Mn 30 mg, Fe 65 mg, Co 0.1 mg, Cu 10 mg, Zn 25 mg, Se 0.1 mg and I 0.5 mg per kg of ration.

### 2.2 Feed, rumen fluid, and muscle sampling and measurement

At the beginning and end of the experiment, 200 g samples were taken from the experimental diet using the quadrant method. The samples were then ground using a grinder to a particle diameter of 1 mm for routine feed analysis. The crude protein content in the feed was measured using the Kjeldahl method. Calcium and phosphorus levels were determined following the Association of Official Analytical Chemists (AOAC) methods (AOAC, 2023). The concentrations of neutral detergent fibre (NDF) and acid detergent fibre (ADF) were assessed according to the method by Hall and Mertens (2023). Rumen fluid samples were collected using a rumen fluid collection tube from the rumen, filtered through four layers of gauze, followed by aliquoting the filtrate into centrifuge tubes. The samples were quickly placed in liquid nitrogen for preservation. Five rumen fluid samples were randomly selected from each group for 16S rRNA high-throughput sequencing analysis. Yaks were slaughtered according to the Chinese “Operational Procedures for Cattle Slaughter” GB/T 19477-2004. After slaughter, a 200 mg sample of the longest dorsal muscle was immediately collected from the right side of the carcass at the 12th-13th rib and stored in sterilized freezing tubes. Five samples from each treatment group were randomly selected for non-targeted metabolomics sequencing.

### 2.3 Meat quality assessment

#### 2.3.1 pH

The pH of the meat was measured following the method described by Huang et al. (2022). Each longest dorsal muscle sample was measured twice: the first measurement was taken 45 minutes post-slaughter, with three readings per sample averaged; subsequently, the samples were placed in a 0~4°C aging room for 24 hours before the second measurement, which was also averaged from three readings.

#### 2.3.2 Meat color

After slaughter, the cut yak meat samples were hung in the aging room for 40 minutes until the color stabilized. A colorimeter was then used to measure the Chroma L*, Chroma a*, and Chroma b* values of the meat samples. To guarantee the precision and consistency of the data, three readings were taken from different regions of each sample, and the mean value was computed.

#### 2.3.3 Shear force

The shear force measurement was conducted according to the Chinese standard “Determination of Meat Tenderness” NY/T 1180-2006. Cooked samples were cooled to a central temperature of -4°C and cut into strips measuring 4 cm× 1 cm× 1 cm along the grain. A shear force measuring device was then used to assess the shear force of the samples. Each piece of meat was measured three times, and the mean value was computed.

#### 2.3.4 Cooking loss

The measurement of cooking loss was conducted following the method described by Li et al. (2023). Longissimus dorsi muscle samples were taken, and surface moisture was removed, with the fascia trimmed off, followed by weighing the samples (recorded as m1). The samples were then placed in cooking bags and heated in a water bath at 80°C for 30 minutes. After removal, the meat samples were allowed to cool to room temperature, and surface moisture was dried again before weighing (recorded as m2). Cooking loss (%) was calculated using the following formula (Equation 1):


(1) $$Cooking\;loss\;\left(\%\right)=\frac{m1-m2}{m1}\times 100\%$$

#### 2.3.5 Water loss rate

The measurement of water loss rate was conducted using the pressure weight method (Warner, 2023). A piece of meat sample was weighed and recorded as m3. The sample was then placed between two layers of gauze, with 16 layers of filter paper in between, and subjected to a pressure of 3.5 MPa for 5 minutes. Afterward, the sample was weighed again and recorded as m4. The water loss rate (%) was calculated using the following formula (Equation 2):


(2) $$Water\;Loss\;Rate\;\left(\%\right)=\frac{m3-m4}{m3}\times 100\%$$

#### 2.3.6 Meat texture

Samples cooled to room temperature were cut into cubes measuring 1 cm× 1 cm× 1 cm. The texture data were measured using a TA3/100 stainless steel cylindrical probe texture analyzer, with the following parameters set: pre-test speed of 2.0 mm/s, test speed of 1.0 mm/s, post-test speed of 1.5 mm/s, compression ratio of 0.5, a 5 s interval between two downward presses, and the mode set to automatic ~20 g. Each piece of meat was tested five times, and the average value was recorded.

### 2.4 Rumen microbiota analysis

Rumen fluid samples from the AL, IR70, and IR40 groups were collected for 16S rRNA amplicon sequencing analysis, which was outsourced to Novogene Corporation Limited in Beijing. The general process included sample preparation, DNA extraction and detection, PCR amplification, product purification, library preparation and quality control, and sequencing using NovaSeq. Total genomic DNA was extracted from the samples using the Cetyltrimethylammonium Bromide (CTAB) method. The library construction was performed using the TruSeq^®^ DNA PCR-Free Sample Preparation Kit. The V3-V4 hypervariable region of the microbial genomic 16S rRNA gene was PCR-amplified using primers 515F (5’-GTGCCAGCMGCCGCGGTAA-3’) and 806R (5’-GGACTACHVGGGTWTCTAAT-3’). After confirming the quality of the amplification products, they were used for subsequent sequencing.

During the analysis, the raw data were processed for merging and filtering to obtain valid data. DADA2 was employed for denoising to generate Amplicon Sequence Variants (ASVs), which were then clustered into Operational Taxonomic Units (OTUs) with 97% similarity. The obtained OTUs were subjected to species annotation to acquire corresponding species information and abundance distribution. Additionally, OTU abundance, Alpha diversity calculations, and Venn diagram analysis were performed to obtain information on species richness and evenness within samples, as well as shared and unique OTUs across different samples or groups. Principal Coordinate Analysis (PCoA) was used to explore differences in community structure between the groups. In the microbial composition analysis, we first used the classify-sklearn algorithm in QIIME2 to classify each amplicon sequence variant (ASV) into species (Bokulich et al., 2018; Bolyen et al., 2019). This classification was based on a feature classifier trained using the Greengenes database, which provided species-level information for subsequent analysis. Once species classification was completed, we conducted differential abundance (DA) analysis to identify significant microbial features between different sample groups. Since microbial data is compositional, traditional statistical methods may not effectively handle this type of data. Therefore, we used the ANCOM-BC tool from the q2-composition plugin in QIIME2 to address biases in compositional data. The ANCOM-BC tool introduces sample-specific bias correction terms, enabling the accurate identification of significant microbial community features between different sample groups. During the analysis, we used the Benjamini-Hochberg (BH) method in the ANCOM-BC tool to adjust the p-values in order to control the false discovery rate (FDR), thereby improving the reliability of the analysis results. Finally, based on the ASV annotation results and the feature table for each sample, we generated species abundance tables at different taxonomic levels (such as phylum, genus, etc.). These abundance tables were then subjected to further statistical analysis. Additionally, we created bar charts of species abundance using Perl 5.26.2 software to provide a more intuitive display of the microbial community composition characteristics.

### 2.5 Untargeted metabolomics analysis of yak longissimus dorsi muscle

The longissimus dorsi muscle samples from the AL, IR70, and IR40 yak groups were analyzed using untargeted metabolomics via LC-MS technology, conducted by Beijing Novogene Technology Company Limited. The process involved grinding the muscle samples in liquid nitrogen, extracting metabolites with 500 μL of 80% methanol-water, followed by centrifugation. After dilution, the supernatant was collected for LC-MS analysis using a Hypesil Gold C18 column and a flow rate of 0.2 mL/min. Data were processed with Compound Discoverer 3.1 for peak alignment, quantification, and metabolite identification through mzVault, mzCloud, and Masslist databases. PCA and OPLS-DA analyses were performed using MetaX, and metabolites were filtered by VIP > 1.0, FC > 1.2 or < 0.833, and *P*-value < 0.05. KEGG and LIPID MAPS databases were used for annotation, and pathway enrichment was analyzed using MetaboAnalyst.

### 2.6 Data statistical analysis

Data were organized using Excel 2021 software and then statistically analyzed using SPSS 27.0 software. Prior to analysis, normality and homogeneity of variance tests were conducted. The Shapiro-Wilk test was used for normality testing, and Levene’s test was used for homogeneity of variance. If the data did not meet normal distribution, logarithmic transformation or Box-Cox transformation was applied to improve normality. If normality was still not achieved after transformation, or if the homogeneity of variance test yielded a significant result (*P* < 0.05), non-parametric tests (e.g., Kruskal-Wallis H test) or Welch-corrected one-way ANOVA were used. For data that meet the assumptions of normality and homogeneity of variance, the LSD method was used for multiple comparisons. If these assumptions were not met, the Dunn test was applied for non-parametric multiple comparisons. To control the false positive rate in multiple comparisons, Holm-Bonferroni correction was used to adjust the significance levels. The specific method is as follows: all p-values from the comparisons were sorted in ascending order, and for the i-th p-value, the adjusted significance level was determined as α/(k−i+1), where k is the number of comparisons and α is the overall significance level (set to 0.05). Through stepwise comparison, the overall false positive rate is kept within the α threshold. Results are presented as means with standard errors of the mean (SEM), with *P* < 0.05 indicating significant differences and *P* < 0.01 indicating highly significant differences. To visually display the similarities and differences between the rumen microbiota samples of the three yak groups, Principal Coordinate Analysis (PCoA) was performed using the “ggplot2” package in R, and corresponding visual plots were generated. To identify differences in the metabolites of the longest dorsal muscle among the three yak groups, orthogonal partial least squares discriminant analysis (OPLS-DA) was carried out using Simca 14.1 software, and visual plots were generated. Subsequently, volcano plots were generated using the “ggplot2” package in R to visually identify metabolites with significant changes and statistical significance. For these significantly different muscle metabolites and rumen microorganisms, Spearman’s rank correlation analysis was performed, and visual plots were created using Cytoscape v3.9.0 software. In the plots, “*” represents *P* < 0.05, and “**” represents *P* < 0.01.

## 3 Results

### 3.1 Effects of different dietary nutritional levels on yak growth performance

The final body weight of yaks in the AL group was significantly higher than that of the IR40 group (*P* < 0.05); the ADG of yaks in the AL and IR70 groups was significantly higher than that of the IR40 group (*P* < 0.05); the dry matter intake (DMI) among the three dietary treatment groups was significantly different (*P* < 0.001)(**Supplementary Table S1**).

### 3.2 Meat quality parameter analysis

From **Table 2**, it is evident that the pH values of the longissimus dorsi muscle at 45 minutes post-mortem differed significantly among the AL, IR70, and IR40 groups (*P* < 0.01). At the 24-hour mark, the pH of the AL group was significantly higher compared to the IR40 group (*P* < 0.05). The Chroma L* value in the AL group was significantly greater than in the IR40 group (*P* < 0.01) and also higher than in the IR70 group (*P* < 0.05). Furthermore, the Chroma a* value was significantly elevated in the AL group compared to the IR40 group (*P* < 0.05). Additionally, the shear force of the longissimus dorsi muscle in the AL group was significantly lower than in the IR40 group (*P* < 0.05). No significant differences were found between the three groups for Chroma b*, cooking loss, water loss rate, hardness, elasticity, cohesiveness, and chewiness (*P* > 0.05).

**Table 2:** Effects of different dietary nutritional levels on the longissimus dorsi muscle in yaks.

Items	AL	IR70	IR40	SEM	P-value
pH_45min_	6.71^a^	6.67^b^	6.47^c^	0.21	<0.001
pH_24h_	5.65^a^	5.57	5.53^b^	0.17	0.021
Chroma L*	32.08^a^	30.75^b^	29.67^c^	0.25	<0.001
Chroma a*	11.58^a^	10.90	10.00^b^	0.26	0.041
Chroma b*	7.94	7.24	7.41	0.18	0.268
Shear force(N)	31.55^a^	36.04	40.83^b^	1.53	0.043
Cooking loss(%)	37.43	36.49	38.32	0.31	0.192
Water loss rate(%)	22.87	24.04	22.04	0.49	0.066
Hardness(g)	3551.00	3675.80	3962.93	189.07	0.670
Adhesion(g)	1658.07	1683.47	1877.80	92.05	0.577
Springiness(mm)	3.23	2.95	2.99	0.78	0.284
Chewiness(mJ)	46.67	49.31	54.43	2.59	0.471

Different lowercase letters above the peer data indicate significant differences (*P* < 0.05), whereas identical letters or no letter indicate no significant difference (*P* > 0.05).

AL: ad libitum feeding group.

IR70: 70% intake group.

IR40: 40% intake group.

### 3.3 Analysis of rumen microbial community composition

**Figure 1:**
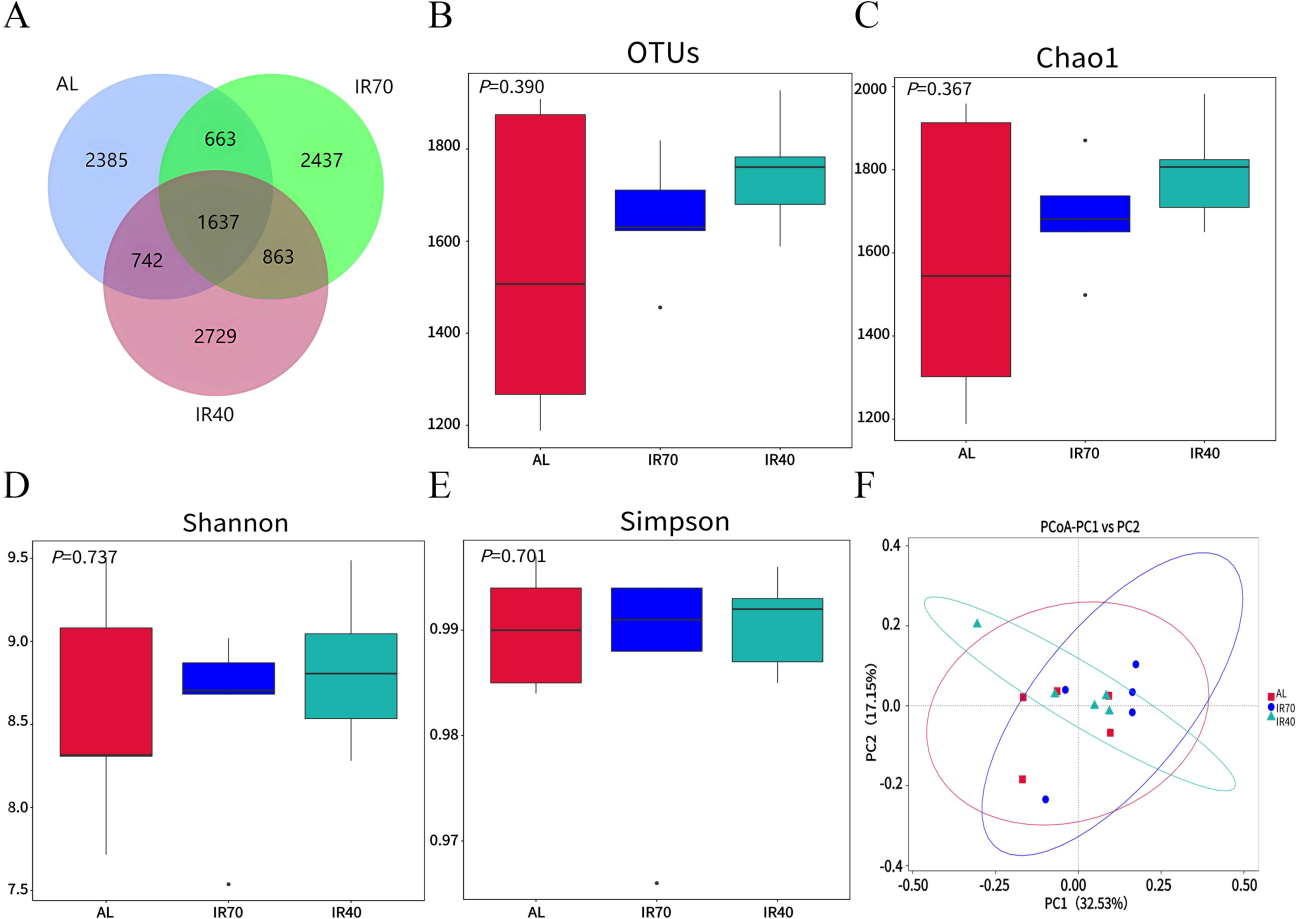
Venn diagram **(A)**. Alpha diversity of rumen bacteria among the three groups **(B–E)**. PCoA analysis **(F)**.

Statistical analysis of the Alpha Diversity indices for different samples showed that the Goods_coverage values were all greater than 0.99, indicating good coverage of the samples. A total of 11 456 ASVs were identified among the three groups, with 1,637 ASVs being common. The specific ASVs observed in the AL, IR70, and IR40 groups were 2 385, 2 437, and 2 729, respectively (**Figure 1A**). No significant differences were observed among the three groups in bacterial richness and diversity, as indicated by OTUs, Chao1, Shannon, and Simpson indices (*P* > 0.05, **Figures 1B–E**). Based on the principal coordinates analysis (PCoA) using Unifrac distance (**Figure 1F**), it was observed that the samples from the three groups tended to cluster together, indicating similar species structure and high community similarity.

**Figure 2:**
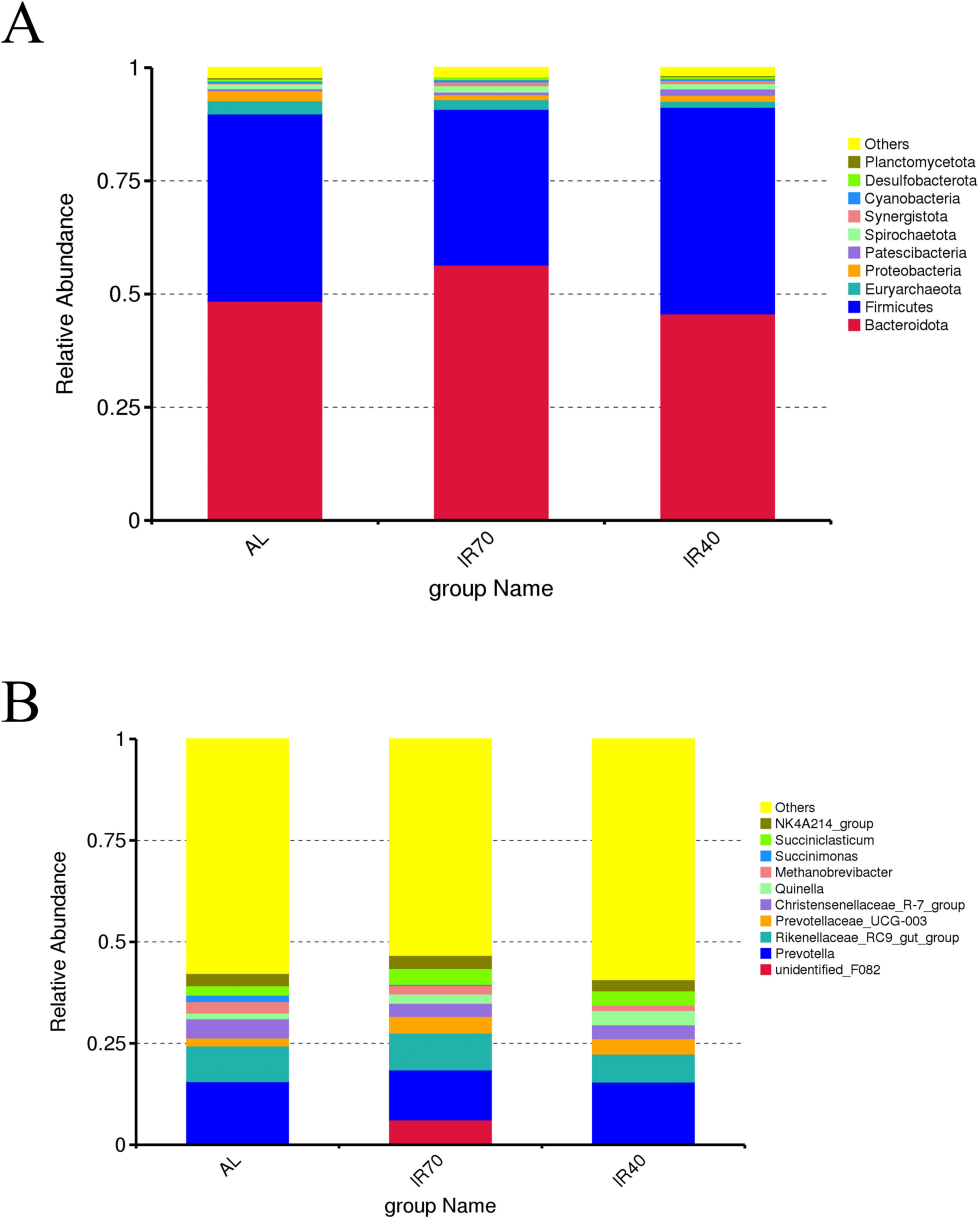
Relative abundance of rumen bacterial phyla **(A)** and genera **(B)**.

At the phylum level (**Figure 2A**; **Supplementary Table S2**), Bacteroidota and Firmicutes were the dominant phyla in the AL, IR70, and IR40 groups, with relative abundances of 50.20% and 40.46%, respectively. The phyla with lower abundances included Euryarchaeota, Proteobacteria, Patescibacteria, Spirochaetota, Synergistota, Cyanobacteria, Desulfobacterota, and Planctomycetota. The AL group exhibited a higher abundance of Bacteroidota compared to the IR70 and IR40 groups, while Firmicutes abundance was lower; however, these differences were not statistically significant (*P* > 0.05). Conversely, the IR40 group showed a significantly greater abundance of Patescibacteria than both the AL and IR70 groups (*P* < 0.05).

At the genus level (**Figure 2B**; **Supplementary Table S3**), *Prevotella* was the most dominant genus (14.28%), followed by Rikenellaceae*_RC9_gut_group* (8.24%), Christensenellaceae*_R-7_group* (3.78%), Prevotellaceae*_UCG-003* (3.31%), *Succiniclasticum* (3.27%), *NK4A214_group* (3.00%), *Quinella* (2.42%), *unidentified_F082* (2.12%), *Methanobrevibacter* (2.06%), and *Succinimonas* (0.69%). The IR70 group had a significantly higher abundance of *unidentified_F082* compared to the AL and IR40 groups (*P* < 0.05). Similarly, *Succinimonas* abundance was significantly greater in the AL group than in the IR70 and IR40 groups (*P* < 0.05).

### 3.4 Analysis of muscle untargeted metabolome

**Figure 3:**
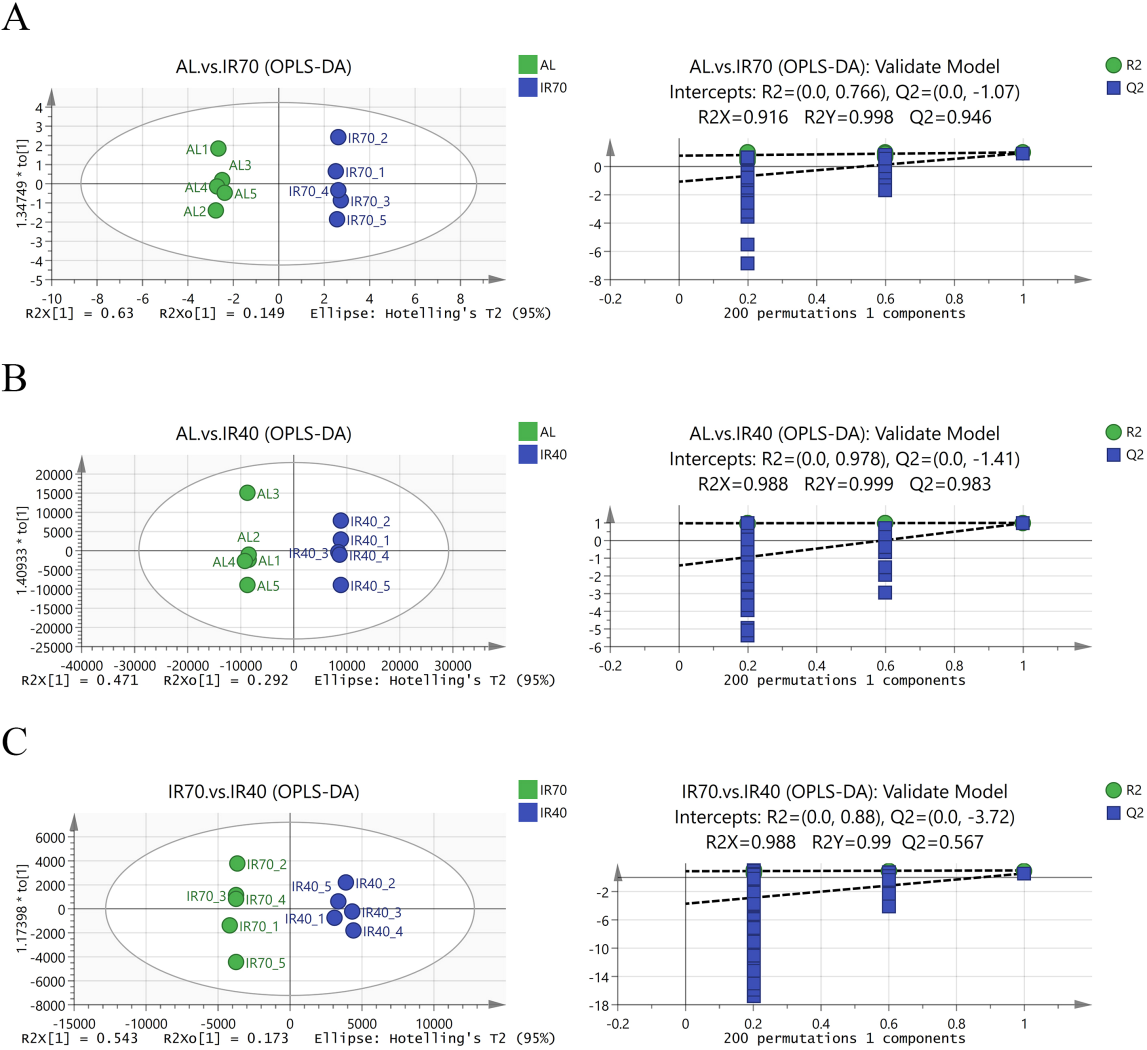
OPLS-DA score plots between the AL group, IR70 group, and IR40 group and the model validation plots **(A–C)**.

Orthogonal Partial Least Squares Discriminant Analysis (OPLS-DA) score plots were generated to illustrate the differential metabolites across the three groups. In the model validation plots for AL group vs. IR70 group (**Figure 3A**), AL group vs. IR40 group (**Figure 3B**), and IR70 group vs. IR40 group (**Figure 3C**), the R^2^X values were 0.916, 0.988, and 0.988, respectively, while the R^2^Y values were 0.988, 0.999, and 0.99. All Q^2^ values exceeded 0.5, suggesting that the model had a good fit and the validation was dependable. The score plots clearly show significant separation and distinction between the different groups, indicating that the OPLS-DA model can effectively identify the differences among the three groups.

**Figure 4:**
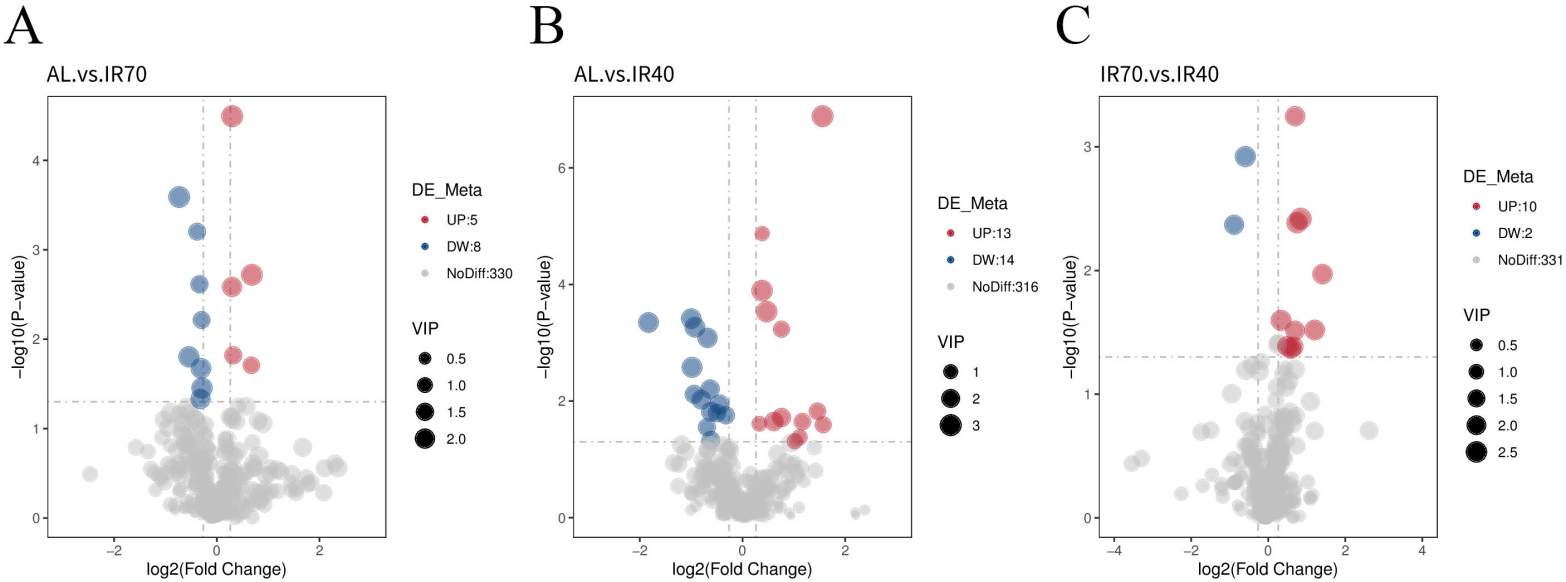
Volcano plots comparing differential metabolites between the AL group and the IR70 group **(A)**, the AL group and the IR40 group **(B)**, and the IR70 group and the IR40 group **(C)**.

The volcano plot visually displays the overall distribution of differential metabolites. The results, shown in **Figure 4** and **Supplementary Table S4**, indicate that a total of 607 metabolites were identified among the 15 samples analyzed. After screening for significantly different metabolites, it was found that there were 13 differential metabolites between the AL group and the IR70 group, with 5 metabolites upregulated and 8 downregulated. In the comparison between the AL group and the IR40 group, 27 metabolites showed significant differences, with 13 upregulated and 14 downregulated. Additionally, there were 12 significant differential metabolites between the IR70 group and the IR40 group, with 10 upregulated and 2 downregulated.

**Figure 5:**
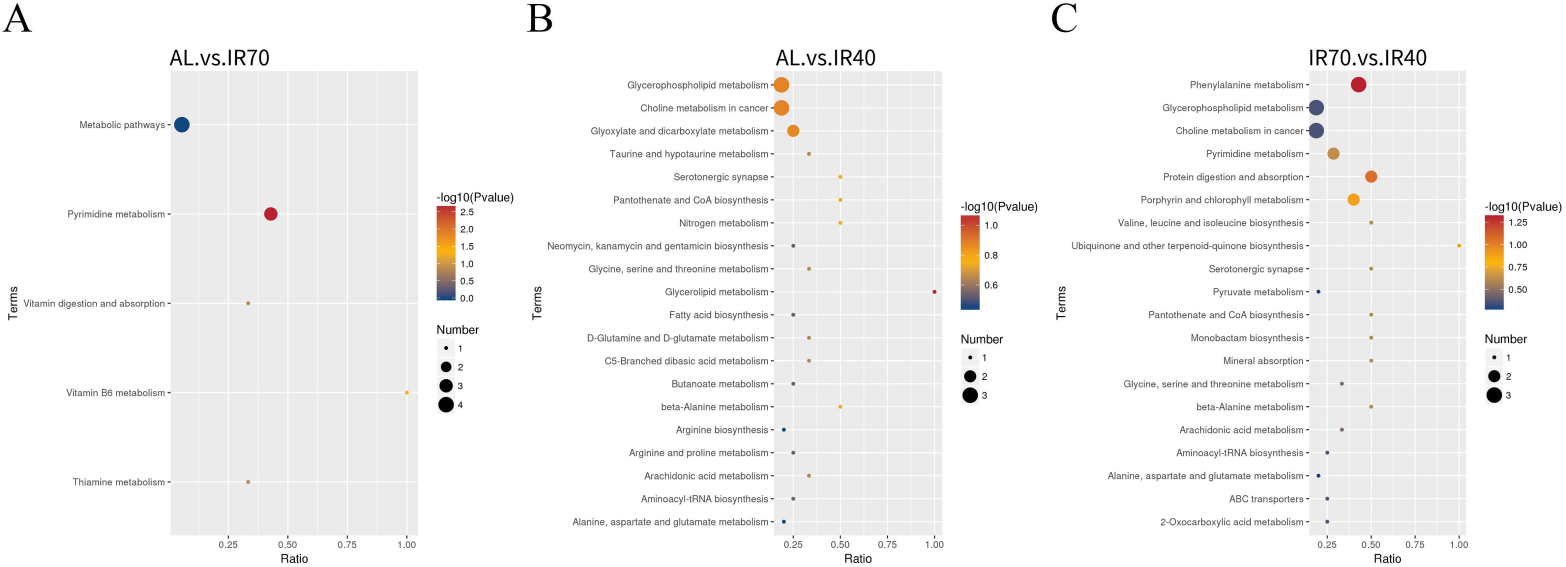
The top 20 enriched KEGG pathway analyses comparing the AL and IR70 groups **(A)**, AL and IR40 groups **(B)**, and IR70 and IR40 groups **(C)**.

According to **Figure 5** and **Supplementary Table S5**, the differential metabolites between the AL group and the IR70 group are mainly enriched in the pyrimidine metabolism and vitamin B6 metabolism pathways. The differential metabolites between the AL group and the IR40 group are primarily enriched in pathways such as Glycerolipid metabolism, Glycerophospholipid metabolism, and Choline metabolism in cancer. The differential metabolites between the IR70 group and the IR40 group are mainly enriched in phenylalanine metabolism, protein digestion and absorption, porphyrin and chlorophyll metabolism, Glycerophospholipid metabolism, pyrimidine metabolism, and Choline metabolism in cancer pathways.

### 3.5 Correlation analysis

To further explore the relationships among meat quality, rumen microbiota, and longissimus dorsi metabolites, we conducted a correlation analysis involving meat quality parameters, the relative abundance of specific bacterial genera in the rumen, and the concentrations of differential metabolites in muscle tissue. Ultimately, 11 metabolic pathways with VIP > 1.0 were identified across the three comparison groups (**Figure 5**), and 37 differential metabolites were selected for further in-depth analysis.

#### 3.5.1 Correlation analysis of meat quality parameters and muscle metabolites at different nutritional levels

**Figure 6:**
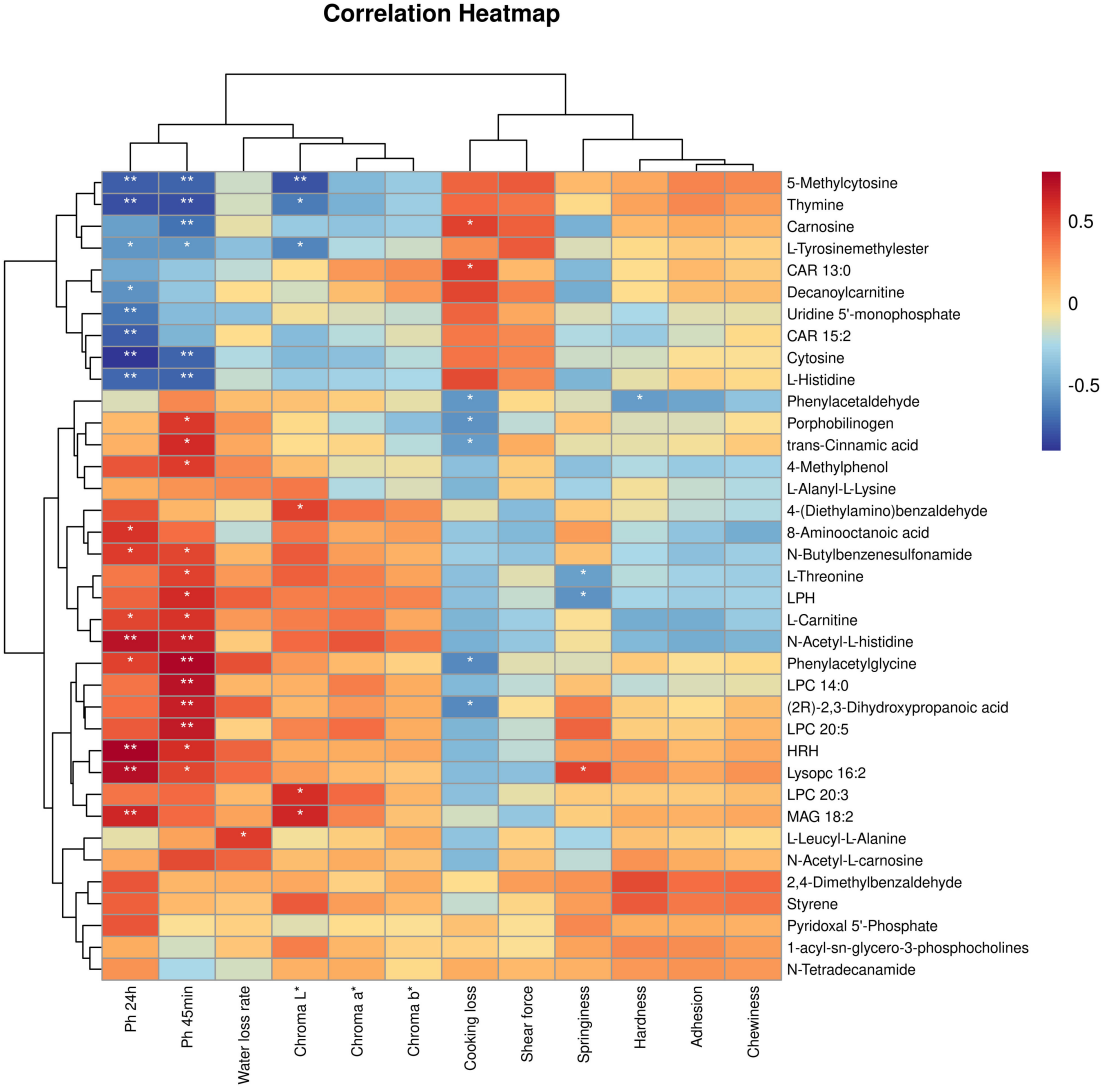
Spearman correlation analysis was performed between the quality parameters of yak longissimus dorsi muscle and the differential metabolites (red denotes a positive correlation, blue indicates a negative correlation). *P < 0.05, **P < 0.01.

Through Spearman correlation analysis between phenotypic data and untargeted metabolomics (**Figure 6**), the following associations were revealed: The pH at 24 hours post-slaughter in the longissimus dorsi of yak showed a positive correlation with MAG 18:2, Lysopc 16:2, HRH, Phenylacetylglycine, N-Acetyl-L-histidine, L-Carnitine, N-Butylbenzenesulfonamide, and 8-Aminooctanoic acid, while it exhibited a negative correlation with Thymine, Cytosine, L-Tyrosinemethylester, L-Histidine, Uridine 5’-monophosphate, CAR 15:2, Decanoylcarnitine, and 5-Methylcytosine. The pH at 45 minutes post-slaughter was positively correlated with Lysopc 16:2, HRH, LPC 20:5, (2R)-2,3-Dihydroxypropanoic acid, LPC 14:0, Phenylacetylglycine, N-Acetyl-L-histidine, L-Carnitine, LPH, L-Threonine, N-Butylbenzenesulfonamide, 4-Methylphenol, trans-Cinnamic acid, and Porphobilinogen, while it exhibited a negative correlation with Thymine, Cytosine, L-Tyrosinemethylester, L-Histidine, and Uridine 5’-monophosphate. The water loss rate was positively correlated with L-Leucyl-L-Alanine. The Chroma L* was positively correlated with MAG 18:2, LPC 20:3, and 4-(Diethylamino) benzaldehyde, while negatively correlated with Thymine, L-Tyrosinemethylester, and 5-Methylcytosine. The Chroma b* was negatively correlated with 5-Methylcytosine. The shear force was negatively correlated with 5-Methylcytosine. Cooking loss was positively correlated with CAR 13:0 and Carnosine, while negatively correlated with Phenylacetaldehyde, Porphobilinogen, trans-Cinnamic acid, Phenylacetylglycine, and (2R)-2,3-Dihydroxypropanoic acid. The springiness was positively correlated with Lysopc 16:2 and negatively correlated with L-Threonine and LPH. The hardness was negatively correlated with Phenylacetaldehyde.

#### 3.5.2 Correlation analysis between rumen bacterial genera and muscle metabolites under different nutritional levels

**Figure 7:**
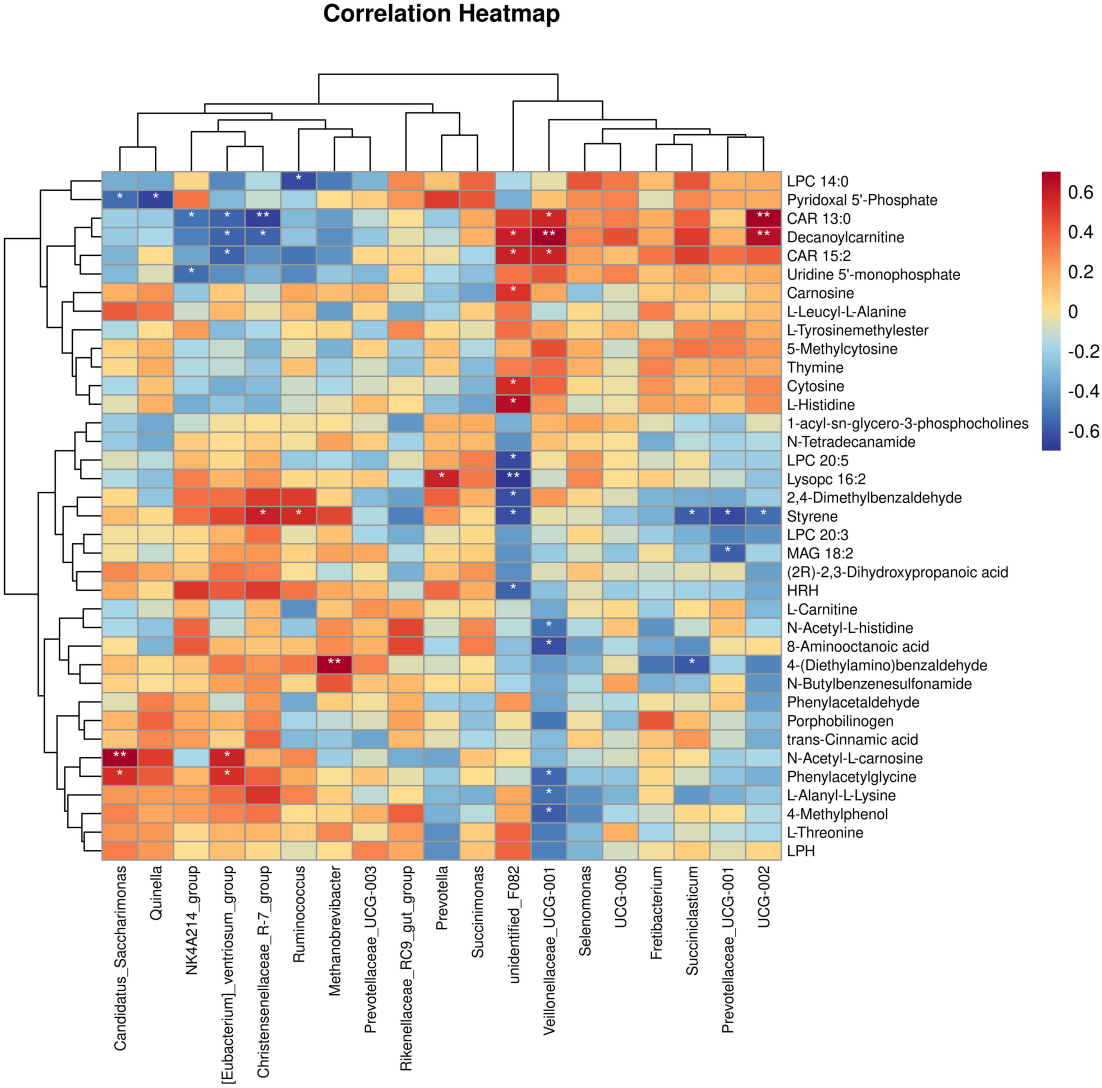
Spearman correlation analysis between the metabolite differences of yak rumen bacteria and the longest back muscle(red denotes a positive correlation, blue indicates a negative correlation). *P<0.05, **P<0.01.

Through the Spearman correlation analysis between the rumen bacterial genera of yaks and the differential metabolites of the longissimus dorsi (**Figure 7**), the following associations were revealed: Veillonellaceae*_UCG-001* was positively correlated with CAR 13:0, CAR 15:2, and Decanoylcarnitine, and negatively correlated with 8-Aminooctanoic acid, N-Acetyl-L-carnosine, 4-Methylphenol, L-Alanyl-L-Lysine, and Phenylacetylglycine. unidentified*_F082* was positively correlated with CAR 15:2, Decanoylcarnitine, L-Histidine, Cytosine, and Carnosine, and negatively correlated with Lysopc 16:2, LPC 20:5, HRH, 2,4-Dimethylbenzaldehyde, and Styrene. *Succiniclasticum* was negatively correlated with 4-(Diethylamino)benzaldehyde and Styrene. Prevotellaceae*_UCG-001* was negatively correlated with MAG 18:2 and Styrene. *UCG-002* was positively correlated with Decanoylcarnitine and CAR 13:0, and negatively correlated with Styrene. *Prevotella* was positively correlated with Lysopc 16:2. Candidatus*_Saccharimonas* was positively correlated with N-Acetyl-L-histidine and Phenylacetylglycine, while it was negatively correlated with Pyridoxal 5’-Phosphate. *Quinella* was negatively correlated with Pyridoxal 5’-Phosphate. *NK4A214_group* was negatively correlated with Uridine 5’-monophosphate and CAR 13:0. [Eubacterium]*_ventriosum_group* was positively correlated with N-Acetyl-L-histidine and Phenylacetylglycine, while negatively correlated with CAR 13:0, CAR 15:2, and Decanoylcarnitine. Christensenellaceae*_R-7_group* was positively correlated with Styrene and negatively correlated with CAR 13:0 and Decanoylcarnitine. *Ruminococcus* was positively correlated with Styrene and negatively correlated with LPC 14:0. *Methanobrevibacter* was positively correlated with 4-(Diethylamino)benzaldehyde.

## 4 Discussion

This study shows that different dietary nutritional levels significantly affect the growth performance and feed intake of yaks. The final body weight of the AL group and the daily weight gain of the IR70 and AL groups were significantly higher than that of the IR40 group. However, it is worth noting that although the IR40 group was fed at the maintenance level, its body weight still increased. This may be attributed to the high fat content in the feed. It is known that a high-fat diet can lead to increased energy intake, which in turn can promote weight gain (Mateusz et al., 2024). The significant difference in dry matter intake (DMI) (*P* < 0.001) further explains this phenomenon, with the AL group having the highest feed intake and the IR40 group having the lowest. This suggests that the nutritional supply of feed directly impacts growth performance.

The pH of the meat is influenced by the process of glycogen glycolysis in the muscle (Ren et al., 2024). Research shows that the pH of animal muscle after slaughter ranges between 6.0 and 7.0, and in the following 1 hour, the pH of the muscle gradually decreases (Lytras et al., 1999). After acidification, the pH at 24 hours ranges from 5.11 to 6.69 (Lou et al., 2024). In this study, both pH at 45 minutes and pH at 24 hours were within this range, indicating that the meat typically exhibits good color, texture, and flavor, making it suitable for further processing and consumption. However, with the decrease in nutritional level, the pH at 45 minutes and pH at 24 hours of the longissimus dorsi muscle in yaks showed a downward trend, which may be related to the rate of glycogen glycolysis in the muscle (Zhu et al., 2024). When the nutritional level is low, the glycogen content in beef decreases, as energy restrictions inhibit glycogen synthesis (Wicks et al., 2019). Chroma is one of the primary factors consumers consider when purchasing beef (Gagaoua et al., 2023). Studies have shown that supplementation can increase Chroma L* (Hao et al., 2020; Wang et al., 2024). In this study, with the increase in nutritional levels, the Chroma L* of the longissimus dorsi muscle in yaks significantly improved. Meanwhile, the Chroma a* of the longissimus dorsi muscle in the AL group was significantly higher than in the IR40 group, suggesting that nutritional levels have a considerable effect on the Chroma of yak longissimus dorsi. Shear force is an important indicator for measuring meat texture, reflecting the force required to cut through the meat. In meat quality evaluation, the magnitude of shear force is directly related to the tenderness of the meat (Al-Moadhen et al., 2024). Shear force is inversely related to meat tenderness, meaning that the more tender the muscle, the lower the shear force value, making it easier to cut (Madhusankha and Thilakarathna, 2021). Studies have shown that animals with high nutritional levels typically have better meat tenderness (Warner et al., 2022). In this study, the shear force of the longissimus dorsi muscle in the AL group was significantly lower compared to the IR40 group, indicating higher tenderness. This aligns with the aforementioned studies, suggesting that increasing nutritional levels within a certain range affects the shear force of the longissimus dorsi muscle in yaks, thereby enhancing beef quality.

Changes in the rumen microbial community help us analyze the effects of different nutritional levels on yaks. The study found that there were no significant differences in the α and β diversity indices among the AL, IR70, and IR40 groups under different nutritional levels, with the OTU, Chao1, Shannon, and Simpson indices of bacterial richness and diversity being similar among the treatment groups. The PCoA plot shows that the samples from the three groups tend to cluster together, indicating that the species structure is similar and the community similarity is high. These results are consistent with those of Zhang et al. (2023), suggesting that host specificity of the rumen microbial community plays an important role in this study (Clemmons et al., 2019). At the phylum level, various studies have demonstrated that Bacteroidota and Firmicutes are the predominant phyla in the rumen bacterial community of ruminants (Bi et al., 2018; Guo et al., 2024), which is consistent with the findings of this study. Firmicutes can degrade organic matter such as cellulose, proteins, polysaccharides, and amino acids through its metabolic activities (Thi Vinh et al., 2024), while Bacteroidota is responsible for breaking down macromolecules of dissolved organic carbon, such as proteins and polysaccharides (Spirito et al., 2018). It has been reported that a high relative abundance of Firmicutes increases the ratio of Firmicutes to Bacteroidota, which is closely related to the efficient utilization of feed by animals (Myer et al., 2015). In contrast, yaks with lower feed utilization efficiency have a higher abundance of Bacteroidota and a lower abundance of Firmicutes in the rumen (Zou et al., 2019). In this study, the relative abundance of Bacteroidota in the AL group was higher than that in the IR70 and IR40 groups, while the relative abundance of Firmicutes was relatively lower. This microbial community characteristic suggests that the feed utilization efficiency in the AL group is relatively lower. Growth performance data further support this conclusion, with the feed-to-weight ratio in the AL group being significantly higher than in the other two groups. This difference in feed utilization efficiency may be associated with the higher proportion of roughage in the AL group’s feed. At the genus level, the research indicated that Succinimonas was considerably more abundant in the AL group compared to the IR70 and IR40 groups. *Succinimonas*, along with other succinate-producing bacteria, is significantly more abundant in the rumen of yaks compared to other ruminants (Zhao et al., 2022). Members of these genera can utilize sugars and other fermentation products from microorganisms to produce succinate, lactate, acetate, and formate (Xue et al., 2022). They do this by participating in carbohydrate metabolism, interacting with other microbes, and adapting to the rumen environment. The high relative abundance of *Succinimonas* in the AL group may be associated with a higher nutritional level. The elevated nutrition level provides more fermentation substrates and energy sources for *Succinimonas*, thereby promoting the proliferation of this microbial group.

In the comparison between the AL group and the IR70 group, the main differential metabolites were enriched in the pyrimidine metabolism and vitamin B6 metabolism pathways. The pyrimidine metabolism pathway is involved in the synthesis of DNA and RNA, as well as other biosynthetic processes. 5-methylcytosine, a methyl derivative of cytosine, can serve as a substrate for DNA repair and recombination, contributing to the maintenance of genomic stability (Stanojević et al., 2024). Thymidine serves as a substrate for RNA synthesis. In addition, scientists have discovered a novel biosynthetic pathway for thymidine. The flavin-dependent thymidylate synthase (FDTS) in this new thymidine synthesis pathway primarily occurs in bacteria and viruses, providing new targets for the research of antibacterial and antiviral drugs (Koehn et al., 2009). The modification status of cytosine can influence cell differentiation. In Th17 cells, the modification of 5-methylcytosine regulates cell differentiation by influencing the activity of transcription factors, thereby impacting immune responses and the progression of inflammatory diseases (Yang et al., 2023). In this study, the levels of several metabolites were significantly upregulated in the IR70 and IR40 groups. This upregulation may be associated with the maintenance of essential metabolic activities and cellular functions, thereby enhancing cell proliferation. Pyridoxal 5’-phosphate, one of the active forms of vitamin B6, serves as a cofactor for enzymes involved in the metabolism of proteins and amino acids. It is essential for maintaining normal physiological processes, including immune function and neural transmission (Gupta, 2022). As a coenzyme, vitamin B6 participates in various enzymatic reactions involved in protein metabolism, including transamination, decarboxylation, β-elimination, β-substitution, γ-elimination, and γ-substitution, among others (Liu et al., 2023). These reactions are directly involved in amino acid metabolism, with amino acids serving as the fundamental building blocks of proteins. Therefore, an increase in the protein content of feed implies a corresponding increase in the demand for vitamin B6 (Dang, 2016), in order to support protein synthesis and catabolism within livestock and poultry. In this study, with the increase in nutritional levels, the content of pyridoxal phosphate in the longissimus dorsi muscle of the AL group was higher than that of the IR70 group. This may be due to the increased protein content in the feed, which raises the demand for vitamin B6 in animals, subsequently resulting in elevated levels of vitamin B6 metabolites in the muscle.

In the comparison between the AL group and the IR40 group, the main differential metabolites were enriched in the glycerolipid metabolism and glycerophospholipid metabolism pathways. (2R)-2,3-Dihydroxypropanoic acid, also known as glyceric acid, is primarily associated with energy metabolism and gluconeogenesis processes in ruminants. In the rumen of ruminants, a significant amount of fibrous materials is fermented by microorganisms, producing volatile fatty acids (VFAs), which serve as the primary energy source for these animals. Among these, 2,3-Dihydroxypropanoic acid acts as an intermediate in gluconeogenesis, participating in the synthesis of glucose within the body. Gluconeogenesis is a process that occurs in the liver, allowing non-carbohydrate substrates (such as certain amino acids, lactate, and glycerol) to be converted into glucose for use by the body (Qingwen et al., 2025). In this study, the content of (2R)-2,3-Dihydroxypropanoic acid in the muscle of the IR40 group was higher compared to the AL group. This suggests that under limited energy intake, yaks may enhance gluconeogenesis and other metabolic adjustment mechanisms to maintain energy balance and protein synthesis, thereby adapting to an environment with insufficient energy supply and maintaining normal physiological function.

In the comparison between the IR70 group and the IR40 group, the main differential metabolites were enriched in the pyrimidine metabolism and protein digestion and absorption pathways. Uracil plays a crucial role in cellular metabolism and is one of the four nucleotides that make up RNA molecules. Uracil participates in encoding genetic information by pairing with other nucleotides and plays a role during the transcription phase of protein synthesis (Orndorff et al., 2023). Threonine is one of the essential amino acids and is crucial for the growth and development of ruminants. The increased levels of uracil and threonine in the muscle tissue of the IR70 group may indicate enhanced protein synthesis activity within the body, which is essential for muscle growth and repair. This response reflects adequate feed intake and a favorable nutritional status (Lv et al., 2022).

Correlation analysis between meat quality phenotypic data and untargeted metabolomics results indicated significant correlations between meat quality parameters and the metabolites in the longissimus dorsi muscle. Previous studies have identified pyrimidine metabolism as a potential biomarker for predicting meat quality (Zhang et al., 2022). In this study, as the nutritional levels increased, the meat’s elasticity gradually improved, while adhesiveness and chewiness showed a decreasing trend. Changes in nutritional levels impact pyrimidine metabolism, resulting in the upregulation of metabolites such as Cytosine, 5-Methylcytosine, and Thymidine. These metabolites, as components of nucleic acid metabolism, provide abundant substrates for the Maillard reaction. Many flavor compounds generated in cooked meat are closely linked to the Maillard reaction (Legako, 2020), and nucleic acid metabolites serve as primary flavor precursors in meat (Kim et al., 2023). Therefore, changes in nutritional levels can alter the flavor of the meat. Cinnamic acid typically exists in the form of its trans isomer, known as trans-Cinnamic acid (Tsuzuki et al., 2024). Cinnamon has garnered attention for its antioxidant and antibacterial properties. Active compounds in cinnamon, such as cinnamaldehyde and phenolic compounds, are used as additives in poultry feed to enhance growth performance, health, and meat quality (Yaqoob et al., 2022). Studies have demonstrated that cinnamon and its components, including trans-cinnamic acid, possess inhibitory effects against various bacteria, including those that have developed antibiotic resistance (Vasconcelos et al., 2018). Incorporating cinnamon essential oil into the feed can significantly influence the pH value of the meat, but it does not affect its sensory or visual acceptability (Torrecilhas et al., 2021), which aligns with the findings of this experiment. Phenylacetaldehyde, as a volatile flavor compound, can be formed in meat products through various pathways, including fat oxidation, the Maillard reaction, and specific microbial metabolic processes (Starowicz and Zieliński, 2019). During the processing of meat products, aldehydes are major potential contributors to the flavor of aged beef. This suggests that benzaldehyde, as one of the aldehyde compounds, contributes to the flavor of beef (Liu et al., 2024).

Rumen bacteria, through their interactions with the host, indirectly influence the deposition of metabolites in the muscles. Among these, *Methanobrevibacter* is a key member of the methanogenic archaea and plays a crucial role in the rumen. They typically utilize hydrogen and carbon dioxide produced by the fermentation of other microorganisms as substrates to generate methane (Mizrahi et al., 2021). 4-(Diethylamino)benzaldehyde is an aldehyde dehydrogenase (ALDH) inhibitor. By inhibiting the activity of ALDH, it can affect the production of hydrogen (Ibrahim et al., 2022). In this study, *Methanobrevibacter* showed a positive correlation with 4-(Diethylamino)benzaldehyde. This suggests that when the relative abundance of *Methanobrevibacter* is higher, the production and utilization of hydrogen in the rumen are more active. 4-(Diethylamino)benzaldehyde can reduce hydrogen production by inhibiting the activity of ALDH, thereby decreasing methane production. Therefore, when the relative abundance of *Methanobrevibacter* is higher, more 4-(Diethylamino)benzaldehyde is required to inhibit ALDH and reduce hydrogen production. This leads to an increase in the concentration of 4-(Diethylamino)benzaldehyde metabolites in the muscles. Candidatus*_Saccharimonas* is positively correlated with metabolites involved in amino acid biosynthesis and energy substrate metabolism (Ogunade et al., 2019). In the processes of amino acid biosynthesis and energy substrate metabolism, phenylacetylglycine serves as a precursor that can be hydrolyzed by enzymes through microbial action to produce phenylalanine and glycine, providing the essential building blocks for protein synthesis. In this study, Candidatus*_Saccharimonas* was positively correlated with phenylacetylglycine, which may be attributed to an increase in Candidatus*_Saccharimonas* biomass, leading to a rise in the total microbial protein in the small intestine. This increase, in turn, enhances the total amount of absorbable and utilizable amino acids in the small intestine, promoting improved production performance in yaks and thereby increasing the protein content in yak muscle.

## 5 Conclusion

In summary, compared to the IR70 and IR40 groups, the AL group exhibited better meat quality (Chroma L*, Chroma a* and Shear force). At the same time, the relative abundance of *Methanobrevibacter* in the rumen was higher in the AL group. Notably, this bacterium showed a positive correlation with the presence of 4-(diethylamino)benzaldehyde, suggesting that the concentration of this compound in muscle tissue may reflect the relative abundance of *Methanobrevibacter* in the rumen. Additionally, the upregulated levels of metabolites such as cytosine, 5-methylcytosine, thymine, trans-Cinnamic acid and phenylacetaldehyde in the AL group contribute to the development of beef flavor. These findings provide valuable insights into the management of yak husbandry and the molecular regulatory mechanisms behind beef flavor formation. However, the dietary nutritional needs of yaks and the volatile flavor compounds in their meat, along with the specific mechanisms behind their changes, require further research.

## Data Availability

The original contributions presented in the study are publicly available. Sequencing data can be found here: NCBI BioProject, accession PRJNA1187203. All other data will be made available by the authors, without undue reservation.
