# Supplementary material for: The effects of different dietary nutritional levels on meat quality, rumen microbiota, and muscle metabolomics in Tibetan Plateau yaks
Source: Front Microbiomes. 2025 Mar 10;4:1545689. doi: 10.3389/frmbi.2025.1545689 (PMC12993581; doi:10.3389/frmbi.2025.1545689)
Supplement: Supplementary file 1 [file DataSheet1.pdf]

Supplementary Table S1. Growth performance.

**Items**	**AL**	**IR70**	**IR40**	**SEM**	***P*-value**
Initial body weight (kg)	237.58	235.52	228.78	2.758	0.426
Final body weight (kg)	320.20a	310.40	288.80b	4.783	0.010
ADG (g/d)	786.86a	713.14a	571.62b	34.596	0.021
Dry matter intake（kg/d）	4.27a	3.21b	1.68c	0.292	＜0.001
weight-to-material ratio	5.55a	4.53b	2.95c	0.330	＜0.001

a–c means within a row with different subscripts differ when *p*-value < 0.05.

Supplementary Table S2. Effects of different dietary nutritional levels on the phyla of rumen bacteria in yaks.

**Phylum**	**AL**	**IR70**	**IR40**	**SEM**	***P*-value**
Bacteroidota	48.47	56.45	45.68	2.527	0.203
Firmicutes	41.37	34.40	45.62	2.405	0.158
Euryarchaeota	2.90	2.13	1.33	0.693	0.684
Proteobacteria	2.21	1.11	1.34	0.547	0.717
Patescibacteria	0.46a	0.57a	1.41b	0.353	0.042
Spirochaetota	1.05	1.42	1.15	0.161	0.656
Synergistota	0.20	0.82	0.72	0.180	0.355
Cyanobacteria	0.45	0.50	0.37	0.120	0.971
Desulfobacterota	0.45	0.53	0.44	0.074	0.882
Planctomycetota	0.30	0.03	0.25	0.111	0.616
Others	2.15	2.04	1.69	0.182	0.609

a−c means within a row with different subscripts differ when p-value < 0.05.

Supplementary Table S3. Effects of different nutritional levels on the genera of rumen bacteria in yaks.

Genus	AL	IR70	IR40	SEM	*P*-value
unidentified_*F082*	0.17a	6.18b	0.40a	1.662	0.014
Prevotella	15.44	12.31	15.10	1.112	0.484
Rikenellaceae_*RC9_gut_grou*p	8.77	9.09	6.87	1.076	0.694
Prevotellaceae_*UCG-003*	1.98	4.12	3.83	1.015	0.680
Christensenellaceae_*R-7_group*	4.72	3.23	3.40	0.623	0.600
Quinella	1.45	2.29	3.52	0.661	0.469
Methanobrevibacter	2.80	2.11	1.26	0.687	0.689
Succinimonas	1.59a	0.27b	0.22b	0.497	0.017
Succiniclasticum	2.33	3.91	3.57	0.534	0.478
NK4A214_group	3.01	3.23	2.76	0.341	0.221
Others	57.74	53.27	59.26	2.016	0.485

a−c means within a row with different subscripts differ when p-value < 0.05.

Supplementary Table S4. Upregulated and downregulated top ten metabolites in AL vs. IR70, AL vs. IR40, and IR70 vs. IR40 comparisons.

Name	Formula	Molecular Weight	RT [min]	m/z	FC	*P*-value	VIP	Up.Down
			**AL.vsIR70**					
4-[(2-cyclohex-1-enylethyl)amino]-2H-chromen-2-one	C17 H19 N O2	247.157	6.276	270.146	0.602	0.000	2.439	down
Pyridoxal 5'-Phosphate	C8 H10 N O6 P	247.024	2.469	248.031	0.767	0.001	1.411	down
N-Desmethyltramadol	C15 H23 N O2	227.188	6.277	250.177	0.791	0.002	1.492	down
N-Tetradecanamide	C14 H29 N O	227.225	8.859	228.232	0.815	0.006	1.424	down
N-(1-benzothiophen-2-yl)-N'-(2-methylphenyl)urea	C16 H14 N2 O S	304.060	6.256	305.068	0.686	0.016	2.241	down
Styrene	C8 H8	104.063	6.440	105.070	0.808	0.021	2.118	down
2,4-Dimethylbenzaldehyde	C9 H10 O	134.073	6.440	135.080	0.819	0.035	2.301	down
4-(Diethylamino)benzaldehyde	C11 H15 N O	177.115	4.796	178.122	0.803	0.047	1.987	down
Thymine	C5 H6 N2 O2	126.043	1.949	127.050	1.230	0.000	2.487	up
L-Leucyl-L-Alanine	C9 H18 N2 O3	202.132	5.277	203.139	1.608	0.002	2.420	up
Cytosine	C4 H5 N3 O	111.043	4.930	112.051	1.228	0.003	1.984	up
5-Methylcytosine	C5 H7 N3 O	125.059	4.508	126.066	1.249	0.015	1.489	up
2,5-bis(4-hydroxy-3-methoxyphenyl)-3,4-dimethyloxolan-3-ol	C20 H24 O6	382.139	5.762	383.146	1.596	0.020	1.346	up

**AL.vs.IR40**								
N1-(3-chlorophenyl)acetamide	C8 H8 Cl N O	169.025	10.373	170.033	0.501	0.000	2.568	down
2-methyl-2,3,4,5-tetrahydro-1,5-benzoxazepin-4-one	C10 H11 N O2	199.061	10.293	200.068	0.281	0.000	2.606	down
L-Carnitine	C7 H15 N O3	161.105	0.518	162.112	0.526	0.001	2.592	down
N-(1-benzothiophen-2-yl)-N'-(2-methylphenyl)urea	C16 H14 N2 O S	304.060	6.256	305.068	0.623	0.001	2.478	down
N-Acetyl-L-histidine	C8 H11 N3 O3	197.080	0.517	198.087	0.506	0.003	2.694	down
Lysopc 16:2 (2N Isomer)	C24 H46 N O7 P	491.300	8.266	492.308	0.646	0.006	2.156	down
HRH	C18 H28 N10 O4	224.115	5.319	225.123	0.520	0.008	1.993	down
ethyl 4-amino-2-(methylsulfanyl)-1,3-thiazole-5-carboxylate	C7 H10 N2 O2 S2	218.019	9.767	219.026	0.573	0.009	2.254	down
2-(3,4-dihydroxyphenyl)-3,5,7-trihydroxy-6-methyl-4H-chromen-4-one	C16 H12 O7	316.058	5.419	317.065	0.733	0.011	2.376	down
N-Butylbenzenesulfonamide	C10 H15 N O2 S	213.082	6.162	214.089	0.652	0.015	2.248	down
3-(propan-2-yl)-octahydropyrrolo[1,2-a]pyrazine-1,4-dione	C10 H16 N2 O2	196.121	5.271	197.129	0.709	0.016	1.871	down
4-(Diethylamino)benzaldehyde	C11 H15 N O	177.115	4.796	178.122	0.799	0.018	1.824	down
8-Aminooctanoic acid	C8 H17 N O2	159.126	2.016	160.133	0.619	0.028	1.798	down
MAG (18:2)	C21 H38 O4	354.275	8.240	355.283	0.652	0.047	2.003	down

2,5-bis(4-hydroxy-3-methoxyphenyl)-3,4-dimethyloxolan-3-ol	C20 H24 O6	382.139	5.762	383.146	2.947	0.000	3.045	up
L-Histidine	C6 H9 N3 O2	155.070	3.110	156.077	1.305	0.000	1.046	up
Thymine	C5 H6 N2 O2	126.043	1.949	127.050	1.302	0.000	2.876	up
Cytosine	C4 H5 N3 O	111.043	4.930	112.051	1.385	0.000	2.885	up
6,7,8-trimethoxy-3-phenyl-2-thioxo-1,2,3,4-tetrahydroquinazolin-4-one	C17 H16 N2 O4 S	344.079	6.518	345.087	1.692	0.001	1.514	up
Decanoylcarnitine	C17 H33 N O4	315.241	6.376	316.248	2.751	0.015	1.637	up
L-Tyrosinemethylester	C10 H13 N O3	195.089	6.461	429.141	1.695	0.019	2.119	up
Carnosine	C9 H14 N4 O3	226.106	5.277	227.114	1.528	0.022	2.163	up
(11E,15Z)-9,10,13-trihydroxyoctadeca-11,15-dienoic acid	C18 H32 O5	345.251	6.235	346.258	2.246	0.023	1.626	up
Uridine 5'-monophosphate	C9 H13 N2 O9 P	324.035	1.594	325.043	1.256	0.025	1.187	up
CAR 13:0	C20 H40 N O4	357.287	7.245	358.295	2.975	0.026	1.512	up
CAR 15:2	C22 H40 N O4	381.288	7.197	382.296	2.162	0.042	1.302	up
1,2-dihydroxyheptadec-16-yn-4-yl acetate	C19 H34 O4	343.272	6.920	344.280	2.027	0.049	1.217	up
				**IR70.vs.IR40**				
L-Threonic acid-1,4-lactone	C4 H6 O4	118.028	1.834	119.035	0.663	0.001	2.429	down

2,5-bis(4-hydroxy-3-methoxyphenyl)-3,4-dimethyloxolan-3-ol	C20 H24 O6	382.139	5.762	383.146	0.542	0.004	2.120	down
LPH	C17 H27 N5 O4	365.206	4.997	366.213	1.625	0.001	2.179	up
N-Acetyl-L-histidine	C8 H11 N3 O3	197.080	0.517	198.087	1.793	0.004	2.698	up
L-Carnitine	C7 H15 N O3	161.105	0.518	162.112	1.678	0.004	2.453	up
2-methyl-2,3,4,5-tetrahydro-1,5-benzoxazepin-4-one	C10 H11 N O2	199.061	10.293	200.068	2.660	0.011	2.222	up
N-Acetyl-L-carnosine	C11 H16 N4 O4	268.116	1.825	269.124	1.260	0.025	2.370	up
L-Alanyl-L-Lysine	C9 H19 N3 O3	217.142	1.243	218.150	2.311	0.030	2.309	up
N1-(3-chlorophenyl)acetamide	C8 H8 Cl N O	169.025	10.373	170.033	1.622	0.031	2.041	up
1-methyl-1H-benzimidazole-2-sulfonic acid	C8 H8 N2 O3 S	212.029	5.230	213.037	1.409	0.041	2.155	up
ethyl 4-amino-2-(methylsulfanyl)-1,3-thiazole-5-carboxylate	C7 H10 N2 O2 S2	218.019	9.767	219.026	1.580	0.041	2.041	up
L-Leucyl-L-Alanine	C9 H18 N2 O3	202.132	5.277	203.139	1.506	0.043	2.242	up

Supplementary Table S5. Analysis of KEGG metabolic pathways for the differential metabolites in the longissimus dorsi muscle.

**MapTitle**	**Metabolites**
**AL.vs.IR70**	
Pyrimidine metabolism	5-Methylcytosine; Cytosine; Thymine
Vitamin B6 metabolism	Pyridoxal 5'-Phosphate
AL.vs.IR40	
Glycerolipid metabolism	(2R)-2,3-Dihydroxypropanoic acid
Glycerophospholipid metabolism	LPC 14:0; LPC 20:5; LPC 20:3
Choline metabolism in cancer	LPC 14:0; LPC 20:5; LPC 20:3
**IR70.vs.IR40**	
Phenylalanine metabolism	Phenylacetaldehyde; trans-Cinnamic acid; Phenylacetylglycine
Protein digestion and absorption	L-Threonine; 4-Methylphenol
Porphyrin and chlorophyll metabolism	Porphobilinogen; L-Threonine
Glycerophospholipid metabolism	1-Acyl-sn-glycero-3-phosphocholine
Pyrimidine metabolism	Uridine; Thymidine
Choline metabolism in cancer	1-Acyl-sn-glycero-3-phosphocholine
